# Evaluating Risk: Benefit Ratio of Fat-Soluble Vitamin Supplementation to SARS-CoV-2-Infected Autoimmune and Cancer Patients: Do Vitamin–Drug Interactions Exist?

**DOI:** 10.3390/life12101654

**Published:** 2022-10-20

**Authors:** Radwa Y. Mekky, Noha M. Elemam, Omar Eltahtawy, Yousra Zeinelabdeen, Rana A. Youness

**Affiliations:** 1 Department of Pharmacology and Toxicology, Faculty of Pharmacy, October University for Modern Sciences and Arts (MSA University), Cairo 12622, Egypt; 2 Sharjah Institute for Medical Research (SIMR), College of Medicine, University of Sharjah, Sharjah 27272, United Arab Emirates; 3 Molecular Genetics Research Team (MGRT), Pharmaceutical Biology Department, Faculty of Pharmacy and Biotechnology, German University in Cairo, Cairo 12622, Egypt; 4 Faculty of Medical Sciences, University Medical Center Groningen (UMCG), University of Groningen, Antonius Deusinglaan 1, 9713 AV Groningen, The Netherlands; 5 Biology and Biochemistry Department, School of Life and Medical Sciences, University of Hertfordshire Hosted by Global Academic Foundation, Cairo 12622, Egypt

**Keywords:** COVID-19, vitamins, drug interactions, therapeutics, cancer, autoimmune diseases

## Abstract

**Simple Summary:**

This review brings attention to a crucial yet under-investigated subject which is vitamin–drug interactions. Fat-soluble vitamins such as vitamins A, D, E, and K have been proven to possess many beneficial effects in the treatment and prevention of COVID-19. Moreover, it has been reported that deficiencies of fat-soluble vitamins have been directly linked to COVID-19 infection-related morbidity and mortality, especially in high-risk populations such as cancer and autoimmune patients. However, many vitamin–drug interactions exist between most of the newly COVID-19 FDA-approved medications and fat-soluble vitamins. Hence, this mandates personalizing the COVID-19 treatment protocols, especially for patients who have any deficiency in any of these vital vitamins. Weighing the risk-to-benefit ratio of supplementing any of these fat-soluble vitamins with COVID-19 medications is considered crucial to maximize the therapeutic benefit and decrease the side effects of these drugs.

**Abstract:**

COVID-19 is a recent pandemic that mandated the scientific society to provide effective evidence-based therapeutic approaches for the prevention and treatment for such a global threat, especially to those patients who hold a higher risk of infection and complications, such as patients with autoimmune diseases and cancer. Recent research has examined the role of various fat-soluble vitamins (vitamins A, D, E, and K) in reducing the severity of COVID-19 infection. Studies showed that deficiency in fat-soluble vitamins abrogates the immune system, thus rendering individuals more susceptible to COVID-19 infection. Moreover, another line of evidence showed that supplementation of fat-soluble vitamins during the course of infection enhances the viral clearance episode by promoting an adequate immune response. However, more thorough research is needed to define the adequate use of vitamin supplements in cancer and autoimmune patients infected with COVID-19. Moreover, it is crucial to highlight the vitamin–drug interactions of the COVID-19 therapeutic modalities and fat-soluble vitamins. With an emphasis on cancer and autoimmune patients, the current review aims to clarify the role of fat-soluble vitamins in SARS-CoV-2 infection and to estimate the risk-to-benefit ratio of a fat-soluble supplement administered to patients taking FDA-approved COVID-19 medications such as antivirals, anti-inflammatory, receptor blockers, and monoclonal antibodies.

## 1. Introduction

It was announced that the global pandemic COVID-19 was caused by the severe acute respiratory syndrome coronavirus 2 (SARS-CoV-2). In December 2019, Wuhan city in China reported the very first reported cases of this virus [1]. Infection with this virus affects leukocyte counts and raises plasma inflammatory cytokines, which results in dysregulation of the immune system [1]. The deregulation in the immune system caused by COVID-19 was the trigger that brought more attention towards cancer and autoimmune patients, due to the critical repercussions associated with the diseases [1].

The relationship between autoimmunity and cancer results from a shared immune system dysregulation, which subsequently raises the risk of infections and other consequences [2,3,4]. The immune status of patients of both diseases is the major issue associating them with a higher susceptibility in acquiring SARS-CoV-2 as well as a more aggressive infection requiring hospitalization due to the complications [5,6]. Additionally, during the pandemic, cancer patients’ mortality rates were found to be greater than those of healthy individuals [7,8]. Inflammatory cytokines unquestionably contribute to an increase in cancer and autoimmune patients’ mortality rates; hence, a more effective treatment strategy is required to suppress immune system hyper-activation and reduce cytokine production [9]. 

Recent research has looked at how several fat-soluble vitamins can reduce the overall severity of COVID-19 infection. The total risk of morbidity and mortality in SARS-CoV-2-infected individuals was found to be reduced as a result of strong evidence that vitamins A, D, E, and K hold a significant role in boosting the defensive mechanism and reducing the cytokine storm and other inflammatory reactions [10]. It is also worth mentioning that vitamin A and D supplementation have recently shown direct and positive effects on the prognosis of virally infected patients, and a tangible improvement was observed in HPV and HIV patients in particular [11,12]. Nonetheless, it has been recently reported that on the molecular level vitamin D has shown a vital modulatory role in tuning functional immunoregulatory features, synchronization between the cellular and viral factors, initiation of autophagy and apoptosis, and even regulating several genetic and episodes during the course of the viral infection [13].

Nonetheless, several studies revealed a link between vulnerability to infection and worse prognosis in COVID-19 patients with deficiencies in one or more of these fat-soluble vitamins [14]. Various new therapeutic interventions have acquired FDA approval or are under clinical trials, including repurposed drugs, antivirals, anti-inflammatory, and immuno-modulatory agents. However, scarce data exist regarding the drug–drug interactions of these vitamins with the new suggested/approved therapeutic drugs. In this review, the authors aim to give a systematic overview of all published data regarding the role of fat-soluble vitamins in SARS-CoV-2, with a special focus on vitamin–drug interactions between the fat-soluble vitamins and the newly approved FDA drug agents for COVID-19 treatment in an effort to outline an effective therapeutic regimen for cancer and autoimmune patients infected with COVID-19.

## 2. Methodology

**Figure 1:**
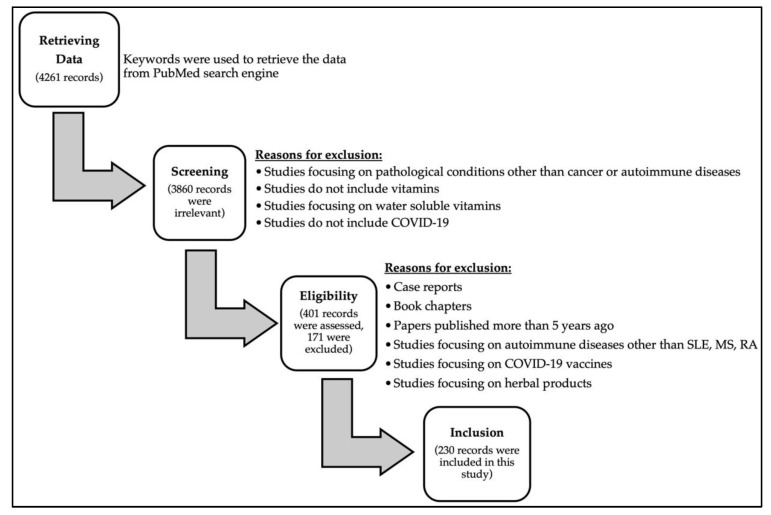
Flowchart diagram outlining the search strategy from initial search to included studies in this review.

The authors aimed at exploring the effects of fat-soluble vitamins in cancer and autoimmune diseases and their link to COVID-19 infection, as well as the vitamin–drug interaction in COVID-19 infection. The authors screened the National Library of Medicine (PubMed). To search databases, the descriptors or keywords used were: “SARS-CoV-2”, “COVID-19”, “Vitamin A”, “Vitamin D”, “Vitamin E”, “Vitamin K”, “Cancer”, “Autoimmune Disease”, “Drug-Drug Interactions”, “Vitamin-Drug Interactions”, “Clinical Trials”, “Systemic Lupus Erythematosus”, “Multiple Sclerosis”, “Rheumatoid Arthritis”, and “Fat-soluble Vitamins”, to cover as many articles as possible in the literature. Relevant publications with detailed information were included includingresearch articles, review articles and book chapters were evaluated and summarized in order to fulfill the purpose of this review article. A summary of the search strategy from initial search, data retrieval, and screening for eligible studies to the final included studies is illustrated in Figure 1.

## 3. Insight on Patients with Cancer and Autoimmune Disease during the Pandemic

Cancer patients are enlisted as a high-risk group in the population by the WHO, due to their susceptibility to complications due to their immune-compromised status [15]. However, further classifications of cancer patients according to their current clinical status were made to establish valid connections between their status and risk of contracting COVID-19, which is further summarized in Table 1 [5,16,17].

Reduced T cell counts were considered a characteristic of late-stage cancer patients infected with the virus. Longer prothrombin time, elevated D-dimer and C-reactive protein levels also enhance the risk and mortality of cancer patients when encountering COVID-19 infection [18,19]. Moreover, tumors expressing angiotensin-converting enzyme 2 (ACE2) have been linked to more aggressive COVID-19 consequences in cancer patients, yet studies validating this link are still required [20]. Therefore, the pandemic has worsened the status of cancer patients, thus requiring specific protocols for containing the disease [5]. 

Autoimmune diseases are another class of diseases that have been on the rise and still require deep investigations, due to the comorbidities and mortalities associated with this subclass of diseases [21,22]. Rheumatoid arthritis (RA), multiple sclerosis (MS), and systemic lupus erythematosus (SLE) are among many other autoimmune diseases that have been showing an alarming increase, yet detailed epidemiological studies are still required for their precise monitoring [23,24,25]. The status of autoimmune patients showed controversy in the literature, where Druyan et al. have demonstrated that these patients are not at higher risk during the pandemic, while others reported how certain drugs administered by these patients make them more susceptible to infection [26,27]. The prognosis of these immuno-deregulated patients during the pandemic still requires extensive analysis, specifically for SLE, MS and RA patients [28,29,30].

Further studies emphasizing the link between COVID-19 and autoimmune diseases prognosis is much needed due to the complex heterogeneity of these diseases and the questionable effects of COVID-19 [31]. Moreover, the general assumption that the pandemic negatively affects these groups of the population need to be deeply investigated to provide us with statistically significant relationships and correlations [26]. Thorough analysis of the choice of chronic drugs to be administered during the pandemic is also needed due to the harmful effects that can be induced if patients are on drugs that further aggravate SARS-CoV-2 infection [32,33,34].

## 4. Therapeutic Approaches Targeting COVID-19

There are significant global efforts underway to identify suitable therapeutic modalities to treat COVID-19 infection. One of the quick-acting strategies was drug repurposing, although it had the disadvantage of having poor selectivity and limited value within virus families. Examples of drugs that were repurposed in COVID-19 therapy are listed in Table 2 [35].

The WHO launched many interventional clinical trials of several drugs including antivirals, anticoagulants, anti-inflammatory and some other adjuvant drugs. Remdesivir, an inhibitor of RNA-dependent RNA polymerase, was the first medication to receive FDA approval for use in COVID-19 treatment, especially in emergency circumstances. Later, numerous clinical trials showed that remdesivir inhibits SARS-CoV-2 both in vitro and in vivo [36]. Moreover, in November 2021, the FDA authorized the use of baricitinib, a Janus kinase (JAK) inhibitor, in combination with remdesivir for the treatment of hospitalized COVID-19 patients who need oxygen supplementation [37]. Corticosteroids, in particular, dexamethasone, has been demonstrated to lower mortality rates and decrease disease progression, particularly in individuals with severe COVID-19 [38].

The pulmonary microvascular thrombosis caused by SARS-CoV-2 is thought to be the primary factor causing acute lung injury. Controversial studies exist concerning the use of heparin as an anticoagulant agent for thrombo-prophylaxis against COVID19-associated coagulopathy. This was further supported by a retrospective study, which found that heparin treatment reduced the mortality risk in COVID-19 patients [39]. On the other hand, a clinical trial did not support the prophylactic anticoagulant intake for ICU-admitted COVID-19 patients [40]. The recent guidelines of COVID-19 treatment recommended that anticoagulants and anti-platelets should not be used in non-hospitalized patients [41].

Some other approaches are under clinical investigation to measure their clinical benefit in COVID-19 patients including immunotherapy such as neutralizing monoclonal antibodies and intravenous immunoglobulins. Due to their capacity to regulate the immune response and cytokine storm linked to severe COVID-19, monoclonal antibodies have drawn significant attention in clinical research. One of the main cytokines linked to COVID-19 acute inflammation and the cytokine storm is the interleukin, IL-6.

A decreased mortality rate in COVID-19 hospitalized patients was reported to be associated with the utilization of monoclonal antibody against IL-6 such as tocilizumab [42]. Tocilizumab and dexamethasone have been licensed by the CDC for use in hospitalized patients displaying rapid respiratory decompensation as a result of COVID-19 infection. Another important cytokine is IL-1β, which is crucial in the cytokine storm observed in COVID-19 infection. According to reports, the IL-1β receptor antagonist, anakinra, reduced the hyper-inflammation seen in ICU-admitted COVID-19 patients, hence lowering the mortality rate and reducing the need for invasive ventilation [42]. There are currently more than 30 active clinical trials examining the potential advantages of utilizing anakinra as therapy against COVID-19. IL-17 is an upstream cytokine to IL-1β and IL-6 cytokines production. Monoclonal antibodies against IL-17 such as netakimab were reported to significantly improve the clinical outcome and oxygenation levels through reduction in inflammatory biomarkers in patients with severe COVID-19 [43]. Phase 2 clinical studies for Secukinumab, another IL-17-specific mAb, are currently taking place. Additionally, clinical trials are being conducted to examine the use of monoclonal antibodies called mavrilimumab and lenzilumab against the important pro-inflammatory cytokine, granulocyte-macrophage colony-stimulating factor (GM-CSF), which has been linked to the immune-pathogenesis of COVID-19 [44]. 

Furthermore, monoclonal antibodies against the viral proteins also represent a great promise in COVID-19 treatment. For instance, monoclonal antibodies against the S protein of the virus, an essential protein in viral entry and initiation of host immune responses, block the interaction with ACE2 and consequently prevent viral entry [45]. Recently, the FDA has authorized the emergency use of eight antiviral drugs, including sotrovimab from GSK and Vir Biotechnology, cilgavimab and tixagevimab from Astra-Zeneca, bamlanivimab, and etesevimab from Eli Lilly, and regdanvimab from Celltrion [46]. These anti-viral antibodies showed a significant decrease of 70–85% in hospitalization or death of patients. The CDC’s COVID-19 guidelines [41] advised using bamlanivimab and etesevimab together in patients with mild-to-moderate COVID-19 who are at a high risk of developing a serious illness or even requiring hospitalization [47].

Another immunotherapeutic approach is the use of intravenous immunoglobulin (IVIg) that was used to treat many inflammatory and autoimmune disorders, which includes immunoglobulin G (IgG) obtained from the plasma of healthy donors. Such therapeutic approach could be implemented for the treatment of COVID-19. Although the precise mechanism of how this approach would help to reduce the cytokine storm associated with COVID-19 remains unknown, studies showed that critically ill COVID-19 patients recovered when subjected to high-dose injection of IVIg [48]. Additionally, IVIg may lessen the cytokine storm by scavenging complement components and preventing the activation of innate immune cells [48]. Nevertheless, more evidence is required to weigh the risks and benefits for this therapeutic approach in treatment of COVID-19 patients.

**Table 2:** Classes of repurposed drugs in COVID-19 infection.

Drug Class	Indicated Use	Drug Target	Impact on COVID-19	References
**Antivirals**				
Daclatasvir/Sofosbuvir	HCV	NS5A inhibitor	Decreased the need for ICU and mortality rates	[49]
Danoprevir	HCV	NS3/4A protease inhibitor	Decrease in hospital stay and time to achieve viral clearance	[50]
Favipiravir	Influenza	RdRp inhibitor	Viral clearance in 7 to 14 days and decreased the need for oxygen	[51]
**Anti-inflammatory**				
Imatinib	Cancer	JAK inhibition	- Decreased the duration of mechanical ventilation - Lowered the risk of respiratory failure and death - Chest CT improvement - Lowered the need for ventilator, decreased the severe biomarkers (LDH, CRP and D dimer) - Decrease in time of hospitalization and significant reduction in mortality rate.	[52]
Tofacitinib	RA	JAK inhibition		[53]
Ruxolitinib	RA	JAK inhibition		[54]
Methylprednisolone	Inflammation, immune system disorders	Decrease the proinflammatory cytokines		[55]
Budesonide	Asthma	Inhibition of proinflammatory cytokine production		[56]
Type I interferons	MS	Equilibrates the expression of inflammatory mediators		[57]
**Other drugs**				
Telmisartan	Hypertension	Angiotensin receptor blocker	Anti-inflammatory effects and reduced overall mortality and morbidity	[58]
Bromhexine	Mucolytic	TMPRSS2 protease blocker	Mortality rate lowered when administered early	[59]
Niclosamide	Anti-parasitic	Modification of endosomal pH and inhibition of autophagy as well as virus replication	Decreased the recovery time, especially in patients with comorbidities	[60]

## 5. Personalized Therapeutic Approaches to Cancer Patients Infected with COVID-19 

Several reputable institutions formed specific guidelines that are continuously updated aiming to reduce the negative outcomes and lethality of cancer during the pandemic, such as ESMO and the NCCN [61]. Resuming therapy for cancer patients remained debatable and depended on the ongoing therapeutic approach that varies across cancer types [5]. Likewise, the aggressiveness of cancer in some cases does not allow the patients to stop therapy without the regression of their clinical state [5]. Therefore, the patient’s condition and the disease’s stability determine whether or not the therapy must be discontinued [62].

For stable cancer patients, delaying chemotherapy and surgical interventions is recommended, specifically due to the increased risk of nosocomial transmission through hospitals [62]. For patients where postponement of therapy is not favored, precise precautions in managing cancer patients is vital to prevent further intricate risks of COVID-19 on these highly susceptible patients [62]. Lastly, in case of actually contracting the disease, these patients should be closely monitored and provided with rigorous attention to overcome the possible lethality of the disease [5]. Other treatments have been also discussed such as administration of intravenous immunoglobulins or blood purification therapies which need to be further studied to formulate possible means of combatting the viral infection in these cancer patients [17]. When cancer patients become infected with COVID-19, they receive personalized regimen strategy as shown in Table 3. 

## 6. Personalized Therapeutic Approaches to Autoimmune Patients Infected with COVID-19 

### 6.1. Systemic Lupus Erythematosus (SLE)

SLE patients are generally more susceptible to viral infections and the complications associated with the diseases such as diabetes, hypertension, and obesity, which places them at a stressful position during the pandemic, where their hospitalization and mortality rates exceeded those of healthy individuals [69]. This is due to the fact that SLE induces effects on the body similar to COVID-19, where they both share pro-clotting potential resulting in excessive clotting, which may have dreadful consequences on SLE patients upon contracting SARS-CoV-2 [6,69]. Additionally, T cell activity is usually impaired in SLE patients predisposing them to viral infections. This could explain why infections account for over 30% of SLE patients’ fatalities, with respiratory infections being the most frequent [70]. More investigative analysis is needed to correlate the risk of contraction of SARS-CoV-2 in SLE patients, yet more severe symptoms are most likely to predominate in SLE patients [71]. 

Immunosuppressive drugs dedicated for SLE patients were not contraindicated during the pandemic, according to the American College for Rheumatology (ACR), as they did not appear to affect either the COVID-19 infection cycle or the symptoms [72]. However, the use of steroids in SLE patients is currently controversial, as both the ACR and WHO recommend preventing their usage unless deemed obligatory [32]. However, research revealed that COVID-19-infected SLE patients may benefit from a higher dose of corticosteroids [73]. Several studies showed that hydroxychloroquine was ineffective in preventing the occurrence of COVID-19 [74,75]. Additionally, cardiovascular side effects associated with use of hydroxychloroquine were identified in SLE patients [76]. Hyperpigmentation also was reported in pedatric SLE patients under hydroxychloroquine [77].

### 6.2. Multiple Sclerosis (MS)

MS is another important autoimmune-related neurodegenerative disease with several clinical manifestations that unfortunately result in several varying disabilities in patients [78]. The general connection between MS and elevated risk of infections was linked to poor outcomes of COVID-19 patients due to the nature of the drugs usually prescribed to MS patients including immuno-suppressors [79]. However, this theory has been counteracted with opposing views stating that the low immune status of these patients allows them to avoid the cytokine storm and serious complications related to COVID-19 [80]. The stage and age of the MS patient appear to be connected to the COVID-19 severity, where old age and late stages may be considered risk factors for MS patients [79]. Ocrelizumab (anti-CD20 medication) had a positive effect on MS patients with COVID-19 due to the decreased production of IL-6 by B cells. On the other side, medications such as Alemtuzumab affected CD52, a cell marker for T and NK cells, and worsened MS symptoms in COVID-19 patients [81]. The controversial association between MS and COVID-19 should be further elaborated to develop correct treatment protocols for these patients.

Treatment of MS by disease-modifying treatments (DMTs) has been proven to be a double-edged weapon for patients during this pandemic [82]. Amor et al. have gathered all the usual DMTs and linked them to the risk status of patients, and they have listed which of these drugs can be used during the pandemic and which drugs to discontinue during the course of COVID-19 infection [82]. It is essential to point out that a recent study of 844 MS patients found a strong correlation between COVID-19 severity in MS patients and the use of rituximab or ocrelizumab (anti-CD20 mAbs). The same study also revealed that COVID-19 severity in MS patients was strongly correlated with previous use of methyl prednisolone shortly before encountering COVID-19 infection [83].

An individualized approach should be tailored to each MS patients when being infected with COVID-19 to ensure that highest therapeutic output with least risk of adverse events. MS patients under immunosuppressive agents might have altered and compromised immune system rendering them good candidates to receive antivirals such as monoclonal antibodies against SARS-CoV-2 virus. 

### 6.3. Rheumatoid Arthritis (RA) 

Generally, RA has also been linked to risk of contracting infections and other accompanying disorders, yet the exact role of COVID-19 in aggravating RA is still debatable due to the common effects induced by both [84]. However, RA patients encountering COVID-19 have slightly higher mortality rates than the general population, which makes them more vulnerable to the pandemic [85,86]. It is also important to note that the inflammation state seen in COVID-19 and RA are comparable, leading to the suggestion that various anti-rheumatic medications, including hydroxychloroquine, tocilizumab, baricitinib, and anakinra, could be used as treatment options for COVID-19 infection [87]. On the other hand, the use of certain drugs such as glucocorticoids has been related to more aggressive COVID-19 consequences to these patients [86]. Therefore, the immune status of patients is the major issue associating them with a higher risk in acquiring SARS-CoV-2 and a more aggressive status requiring hospitalization [84].

ACR, National Institute for Health and Care Excellence (NICE), and the Australian Rheumatology Association have provided in-depth analyses on the usage of anti-RA medications and endorsed the use of non-steroidal anti-inflammatory drugs (NSAIDs) in the chronic treatment of RA [88]. In addition, cessation of corticosteroids in patients where these drugs have been chronically prescribed is not recommended. Due to the NICE regulations, new prescriptions to RA patients involving corticosteroids may take place, maintaining the lowest dose possible [88]. Additionally, RA patients currently on immunosuppressives should continue with their use, unless infected with COVID-19, where the treating physician outweighs the benefits and downfalls of treatment cessation [89].

Antiviral therapies such as monoclonal antibodies against SARS-CoV-2 are considered the treatment of choice to RA patients as their immune system is compromised due to co-administration of immune suppressors. Among those monoclonal antibodies are Casirivimab and imdevimab [90]. However, the treatment of RA patients infected with SARS-CoV-2 should be based on the risk factors for poor clinical outcomes, such as the existence of concomitant illnesses, age (>65 years), the clinical severity of COVID-19 infection, and the level of immunosuppression. A customized regimen should be tailored to each individual to maximize the therapeutic benefit. For example, RA patients who are under high dose of immunesuppressants at time of COVID-19 hospitalization would have a higher risk than benefit if administered IL-6, JAK or IL-1 inhibitor as this drugs will increase the possibility of serious hospital-acquired infections [91]. In contrast, RA patients receiving a low dose of methotrexate might be advised to stop methotrexate and receive IL-6, JAK and IL1 inhibitors in combination with dexamethasone. 

## 7. Impact of Fat-Soluble Vitamins in COVID-19 Prevention and Treatment

**Figure 2:**
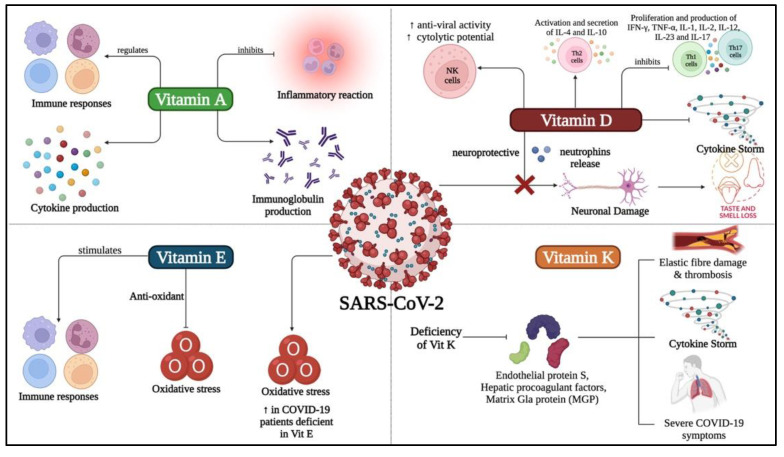
Role of fat-soluble vitamins (vitamin A, vitamin D, vitamin E and vitamin K) in prevention and treatment of COVID-19. Vitamin A was found to regulate immune responses and cytokine production in case of SARS-CoV-2 infection while inhibiting the inflammatory reaction and antibodies production. Vitamin D was found to regulate different types of immune cells and prevents neuronal damage during and post- SARS-CoV-2 infection. On the other hand, Vitamin E was found to initiate the production of antioxidants and stimulates several immune responses in an attempt to eradicate the virus. Vitamin K deficiency was found to be accompanied with severe symptoms of the disease and thrombosis.

Recently nutrients have emerged as the panacea because of their efficacy to treat or prevent disease and exceedingly low risk levels [92,93]. Thus, it makes sense to research their potential curative role for COVID-19. Several reviews have concluded that nutrients are essential to strengthen the immune system throughout a viral infection, especially when infected with SARS-CoV-2 [15,94,95]. With a special focus on vitamins against COVID-19, a recent study has found evidence that supports the use of vitamins to treat COVID-19-like respiratory diseases and requested detailed clinical trials on the effectiveness of vitamins in treating COVID-19 [96,97]. Additionally, studies showed that fat-soluble vitamins are crucial in decreasing the overall severity of COVID-19 infection by alleviating the inflammatory cytokines that causes cytokine storm (Figure 2) [10].

### 7.1. Vitamin A

**Figure 3:**
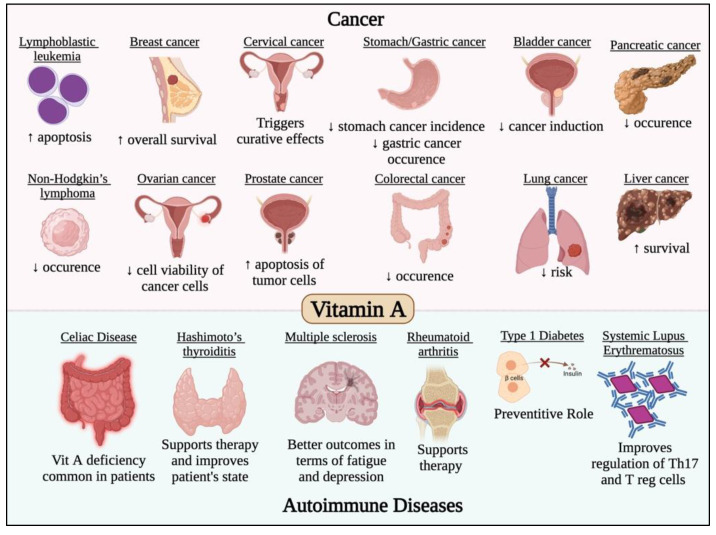
Effect of vitamin A in different cancer types and autoimmune diseases. Vitamin A has potent anticancer activity against different solid and non-solid malignancies such leukemia, lymphoma, breast, cervical, gastric, bladder, ovarian, colorectal, lung, liver and pancreatic cancers. Vitamin A was also found to have potent immunomodulatory roles that would prevent/treat/alleviate the symptoms of several autoimmune diseases such as multiple sclerosis, systemic lupus erythematosus and thyroiditis.

It has long been known that vitamin A possess immunomodulatory roles and is essential for the appropriate performance of the immune system [98]. For example, lack of vitamin A has been linked to a higher risk of death from respiratory infections [98]. Hence, it is essential to investigate the role of vitamin A against COVID-19. A recent review using bioinformatics tools identified the vitamin A associated genes and carried out computational assays to evaluate the effect and mechanism of action of vitamin A against COVID-19 [99]. Generally, its mechanism of action against SARS-CoV-2 consists of several effects such as enrichment of the immune reaction and inhibition of inflammatory reaction. In addition, vitamin A has an effect on a number of cellular responses to the virus, including cytokine release, immunoglobulin synthesis, acute and chronic inflammatory responses, as well as a number of other immunological processes [99]. Further clinical trials are required to explore the importance of vitamin A in the immune system and cellular immunological responses and investigate its therapeutic or preventative effect against COVID-19. This is besides the impact of vitamin A in most cancer types and autoimmune diseases, which has been previously studied and reviewed [100,101,102,103,104,105,106,107,108,109,110,111,112,113,114,115,116,117] and is summarized in Figure 3.

### 7.2. Vitamin D 

**Figure 4:**
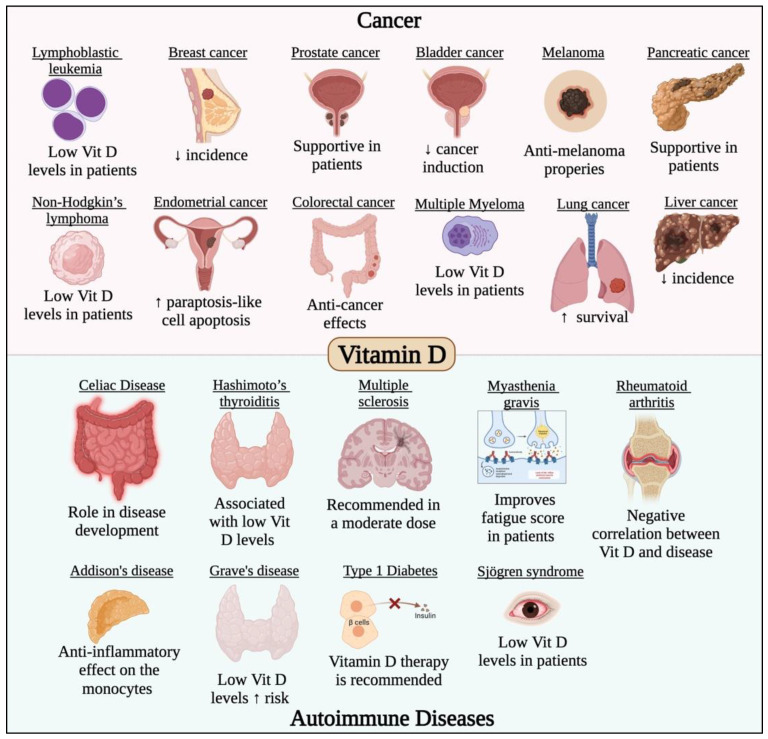
Effect of vitamin D in different cancer types and autoimmune diseases. Vitamin D has potent anticancer activity against different solid and non-solid malignancies such leukemia, lymphoma, breast, prostate, gastric, melanoma, endometrial, colorectal, lung, liver, pancreatic and bladder cancers. Vitamin D was also found to have potent immunomodulatory roles that would prevent/treat/alleviate the symptoms of several autoimmune diseases such as multiple sclerosis, rheumatoid arthritis, and Grave’s disease.

The impact of vitamin D on COVID-19 has been examined in a number of reviews and clinical trials, where vitamin D supplements were reported to lower the risk of infection and could be taken in higher doses in COVID-19 infection [118]. The main role of vitamin D against COVID-19 is being an immunosuppressant that inhibits the cytokine release syndrome which is one of the symptoms of severe COVID-19 [119]. COVID-19 patients suffering from pneumonia showed great benefit from the intake of vitamin D that was found to play a significant effect on the cytokine storm [119]. Moreover, vitamin D prevents Th1 and Th17 cell proliferation and the subsequent release of cytokines including IFN-γ, TNF-α, IL-1, IL-2, IL-12, IL-23, and IL-17 [119]. Additionally, vitamin D functions as a mediator for the differentiation of Th2 cells and the release of their cytokines (IL-4 and IL-10), which are critical in preventing organ damage caused by severe COVID-19 infection [119]. Furthermore, there are several clinical trials investigating the significance of vitamin D in the prevention and treatment of autoimmune diseases because of its immunomodulatory function. Another important role of vitamin D against COVID-19 is its neuroprotective effect [119]. Although headaches and loss of taste and smell are common COVID-19 symptoms, they have not been well investigated. It is possible that SARS-CoV-2 affects the neurons and result in nerve damage leading to the neurological symptoms mentioned above. Moreover, vitamin D regulates the, neurotrophins which are vital for the survival and maintenance of the neurons and promote the expression of brain-derived neurotrophic factor, nerve growth factor, and p75NTR neurotrophin receptor [119]. The immunomodulating role of vitamin D is also considered a mechanism by which it acts as a neuroprotective agent [119]. Additionally, vitamin D3, has been suggested to enhance natural killer (NK) cells’ biological activities, particularly their capacity for cytolysis. These medications may also restore the NK cells’ antiviral activity, thus protecting the COVID-19 infection and its complications [120]. For the reasons mentioned previously and others, many reviews recommend the frequent supplementation of vitamin D to prevent and treat COVID-19 [118,119,121]. Additionally, vitamin D has a critical role in most cancer types and autoimmune diseases, which has been previously studied and reviewed [122,123,124,125,126,127,128,129,130,131,132,133,134,135,136,137] and is summarized in Figure 4.

### 7.3. Vitamin K 

Vitamin K2 and the maintenance of serum 25-hydroxyvitamin D levels have been recently linked to death in COVID-19 patients, as reported by Goddek S., where there was an association of the vitamin levels with calcium deposition in bones, thus increasing its nutritional potential [138]. A previous study linked vitamin K to regressive thrombotic properties and inflammatory cytokine storm in COVID-19 patients where a deficit in the vitamin was a feature of serious COVID-19 cases. This further emphasizes the potential protective role of the vitamin, specifically during the pandemic [139]. Moreover, Janssen et al. further reported the association between this vitamin and aggressive COVID-19 cases due to its role in stimulating thrombogenic properties. Further emphasis on its nutritional positive value was also shown in a region in Japan which is known for a dish rich in vitamin K2 [140]. This was further elaborated where a certain polymorphism involving the low recycling of vitamin K was associated with its advantage during COVID-19 in East Asian populations, providing unique insights on the benefit of the VKORC1-1639A allele in a pandemic setting [140].

**Figure 5:**
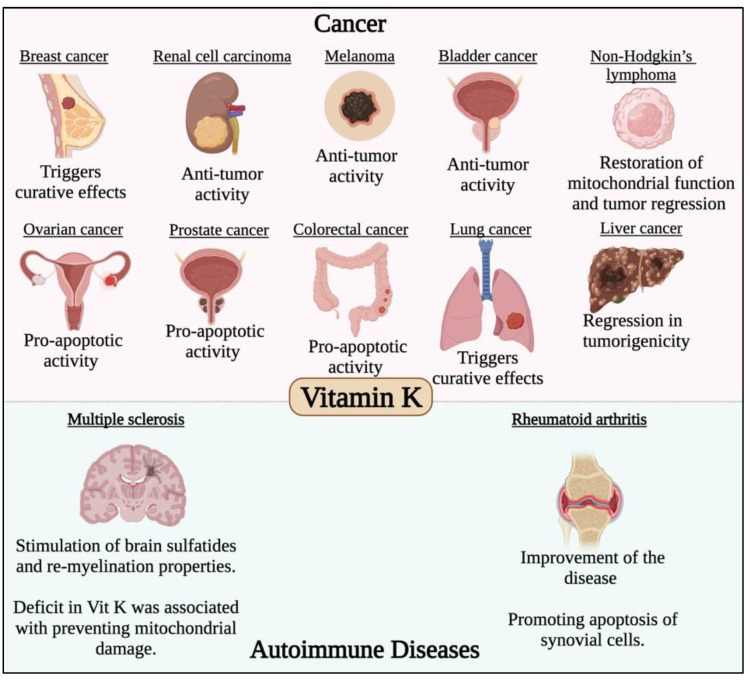
Effect of vitamin K in different cancer types and autoimmune diseases. Vitamin K has potent anticancer activity against different solid malignancies such breast, renal cell carcinoma, melanoma, bladder, ovarian, colorectal, lung, liver and prostate cancers. Vitamin K was also found to have potent immunomodulatory roles in multiple sclerosis and rheumatoid arthritis.

A diminished activation of endothelial protein S and presence of other hepatic procoagulant factors and the matrix Gla protein (MGP), was linked to anti-thrombotic activity and vitamin K insufficiency [141]. A novel relationship between inactive MGP levels and respective vitamin K levels allowed its relation to COVID-19, while the increase in active MGP levels reflected the impact of vitamin K in the inhibition of the lethal effects of the coronavirus and the induction of elastic fibre damage [141]. Additionally, a synergy between vitamin D and vitamin K was necessary, thus necessitating the administration of vitamin K in severe COVID-19 patients prior to vitamin D supplementation [141]. Vitamin K was reported to have positive impacts in most cases of cancer and autoimmune diseases, which has been previously studied and reviewed [142,143,144,145,146,147,148,149,150,151,152,153,154,155,156,157,158] and is summarized in Figure 5. The emerging roles of vitamin K1 and K3 have also been noted due to the notable effects induced by this subclass of vitamins in regulation of tyrosine kinases and their downstream oncogenic pathways, as well as their regulatory effect on certain transcription factors [159]. The exact mechanism underlying its anti-cancer activity was thoroughly discussed by Mamede et al. [160].

### 7.4. Vitamin E

Supplementation of Vitamin E along with vitamins C and D has been stated to be critical to the well-being of individuals as well as those infected with COVID-19 [92]. This goes back to the well-known anti-oxidant properties of vitamin E and its impact on stimulating the immune system and subsequent protection from infections [92]. Further exploration of these immune-boosting properties in COVID-19 patients is currently lacking, yet the general addition of vitamin E to other vitamins carries huge nutritional value [92]. Such properties are much needed by the elderly patients, as their immune responses are known to reduce over time [161]. Additionally, low vitamin E levels in SARS-CoV-2-infected pregnant women was also noted by Erol et al., owing to the oxidative stress induced by the virus, which contributes to negative perinatal effects [96]. Moreover, supplementation of vitamin E to another set of nutrients in obese patients has been linked to more positive outcomes in COVID-19 patients, due its previously mentioned immune-promoting properties [162]. Additionally, an animal-based study aimed to identify the role of vitamin E in Keshan disease, where more favorable outcomes were associated with the availability of the vitamin [163]. A similarly purposed study also related the intake of the vitamin to improved outcomes in mice infected with the influenza virus [164]. The role of vitamin E in human immunodeficiency virus (HIV) was also required to unravel the potential anti-viral effects of its intake [165]. Additionally, the Newcastle disease virus (NDV) is another viral infection where vitamin E was identified to have a great dietary potential due to its regressive effects on the manifestations of the disease [166]. Additionally, the potential anti-viral properties of this vitamin were further demonstrated due to its unique anti-oxidant role with a preventive value in handling recurrent herpes and human papillomavirus [167]. 

**Figure 6:**
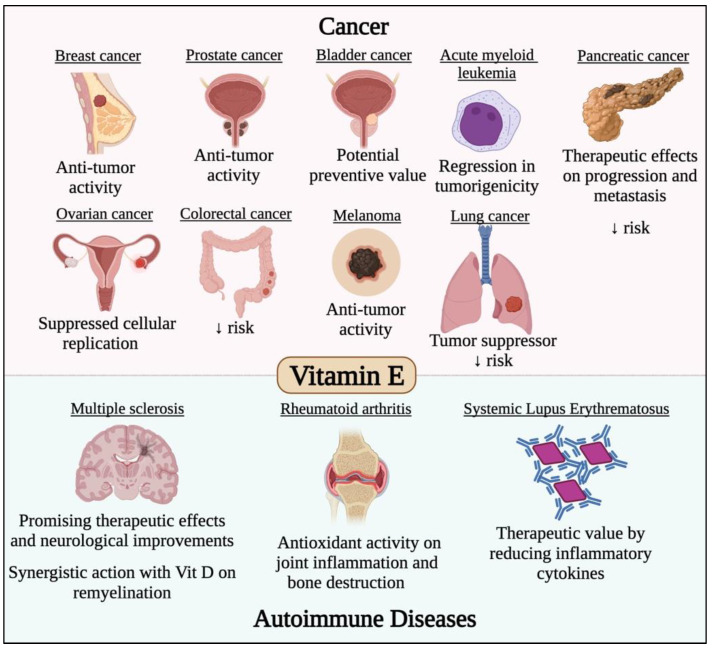
Effect of vitamin E in different cancer types and autoimmune diseases. Vitamin E has potent anticancer activity against different solid malignancies such breast, prostate, bladder, pancreatic, ovarian, colorectal, melanoma and lung cancers. Vitamin E was also found to have potent immunomodulatory roles that would prevent/treat/alleviate the symptoms of several autoimmune diseases such as multiple sclerosis, systemic lupus erythematosus, rheumatoid arthritis and systemic lupus erythematosus.

On the other hand, possible preventive intake of vitamin E was shown to provide beneficial effects on cancer patients due to the positive impacts associated with reduced tumorigenesis in animal models [168]. Moreover, different precursors of vitamin E have been even linked to more predominant anti-carcinogenic effects [169]. However, clinical trials aiming to validate such effects in a clinical setting failed to associate the vitamin with positive effects on cancer patients, which led to a controversy on the role of these potential agents in carcinogenesis [170]. However, isoforms of vitamin E have been recently gaining popularity in the literature, due to their ability to restore the sensitivity of resistant tumors to treatment [171]. The potential effects of vitamin E in various cancer types and autoimmune diseases have been previously investigated [169,172,173,174,175,176,177,178,179,180,181,182,183,184,185,186,187,188,189,190] and are summarized Figure 6.

## 8. Drug–Drug Interactions of Fat-Soluble Vitamins and COVID-19 Therapeutic Agents

The main goal of this review is to evaluate the risk-to-benefit ratio of vitamin supplementation to COVID-19 patients as part of their treatment plan. That is why it is essential to highlight any potential drug interactions between the approved COVID-19 treatment modalities and the four different fat-soluble vitamins.

### 8.1. Drug Interactions of Vitamin A with COVID-19 Therapeutic Agents

As mentioned earlier, vitamin A was identified to exhibit a potential therapeutic effect in COVID-19 patients, where it decreased the incidence of infection and shortened the duration of the disease [191]. Previous studies reported multiple mechanisms of action of vitamin A in infections including enhancement of T cell migration to the thymus as well as promotion of T cell activation and antibody production by B cells, which opens the door to possible interactions of vitamin A and immunomodulatory drugs such as monoclonal antibodies [192,193,194,195,196]. Furthermore, vitamin A is an inhibitor of CYP2C19, which is involved in the metabolism of some drugs involved in COVID-19 treatment [197]. 

Interferon type 1A was a drug that has been postulated to benefit COVID-19 patients among many other viral infections. Vitamin A was known to carry a beneficial role through mounting the therapeutic efficacy of type 1 interferon [198]. Another study suggested that vitamin A has a pivotal role in decreasing the adverse events of some ACE inhibitors [199]. However, many drug interactions still need to be further investigated with this vitamin. 

### 8.2. Drug Interactions of Vitamin D with COVID-19 Therapeutic Agents 

Given that vitamin D may affect the metabolism of many medications that depend on CYP3A4 activation while the CYP3A4 gene contains a vitamin D response element, multiple studies into the vitamin D–drug interactions was deemed essential [200,201]. Given that many of the suggested anti-COVID-19 drug agents are activated/metabolized by CYP3A4, this highlights the importance of studying the drug interactions related to vitamin D [202]. Ivermectin, for instance, has been suggested to have antiviral effect against COVID-19 and is metabolized via CYP3A4, hence a 2 h delay between the administration of ivermectin and vitamin D has been advised [203]. Another medication suggested in COVID-19 therapy due its anti-inflammatory effects is colchicine [204]. Additionally, colchicine is a substrate for CYP3A, and hence, inducers of CYP3A such as vitamin D might lead to a sub-therapeutic dose of colchicine [205]. In contrast, other medications are categorized as CYP3A inhibitors, such as the combination of lopinavir and ritonavir, which is expected to raise the plasma concentrations of any medication that is processed by CYP3A such as vitamin D [205]. This supports another study’s findings, where treatment with lopinavir/ritonavir for 48 weeks dramatically increased serum levels of vitamin D [206]. On the other hand, elevated cytokine levels in COVID-19 are known to downregulate the expression of hepatic enzyme CYP450 [207]. Vitamin D and immunomodulatory agents such as monoclonal antibodies and some antiviral drugs might hold a possible synergistic effect in restoring the CYP450 enzymes [205]. This goes in line with a study that concluded that combinations of vitamin D and remdesivir, enhanced treatment results in the fight against COVID-19 infection [208]. Furthermore, a study demonstrated that the use of monoclonal antibodies as anakinra, infliximab, canakinumab, sarilumab, and tocilizumab restored CYP450 enzyme levels back to normal [205]. Particularly in COVID-19 patients who are continuing to receive therapeutic administration of drugs with a narrow therapeutic index that are substrates for CYP450 enzymes such as warfarin, a potential synergistic effect of these drugs on the restoration of enzymes should be thoroughly investigated.

Previously, the antimalarial drug hydroxychloroquine was used to treat autoimmune diseases such as SLE. Patients receiving hydroxychloroquine had significantly increased levels of vitamin D compared to those who are not taking it [209]. A study found that the activation of CYP3A4 by corticosteroids can lead to a reduction in vitamin D levels [210,211]. On the contrary, another study showed that vitamin D enhanced the efficacy of corticosteroids and attenuated their side effects [212].

### 8.3. Drug Interactions of Vitamin K with COVID-19 Therapeutic Agents

A high mortality rate in COVID-19 infection was significantly associated with the impaired coagulation process. The recent guidelines of COVID-19 treatment recommended that anticoagulants and antiplatelets should not be used in non-hospitalized patients [41]. However, in ICU patients who exhibit coagulopathy, warfarin and heparin could be prescribed to inhibit organ dysfunction and decrease mortality rates [32,41]. Because of its narrow therapeutic index, warfarin is considerably sensitive to variations in the plasma concentrations of vitamin K, and thus, its ability to treat certain conditions is inhibited by high vitamin K intake [213]. However, other controversial studies investigated the interactions of vitamin K and warfarin, where a study concluded that normal daily intake of vitamin K does not affect warfarin efficacy [214]. On the other hand, a study concluded that multivitamin supplements may affect warfarin anticoagulation in susceptible patients [215]. Thus, this debate obliges the need for more studies to investigate the vitamin K dose change in patients administering warfarin. 

### 8.4. Drug Interactions of Vitamin E with COVID-19 Therapeutic Agents

Although vitamin E is well known for its antioxidant properties, a study found a link between excessive vitamin E intake and an increased risk of gastrointestinal cancer, heart failure, and overall cause of death [216]. Moreover, a study concluded that potent antioxidant supplementation such as vitamin E could lower the efficacy of some chemotherapeutic agents such as alkylating agents, platinum compounds, or anthracycline which exert their therapeutic effect by generating active oxygen species [217]. Imatinib is a chemotherapeutic agent that was suggested to be repurposed for COVID-19 therapy. Thus, caution must be taken for cancer patients infected with COVID-19 while administering vitamin E to prevent drug interaction and a decrease in chemotherapeutic efficacy.

P-glycoprotein (P-gp), or multidrug resistance protein 1 (MDR1), is known to increase the efflux of some drugs limiting the intestinal absorption and bioavailability of these drugs. According to several in vitro studies, vitamin E at high concentrations may potentially enhance the activity of P-gp [218]. Lopinavir, ritonavir and sofosbuvir/daclatasvir are among the repurposed drugs suggested for treatment of COVID-19, with their efflux mediated by P-gp [219,220]. Therefore, the enhancement of P-gp with vitamin E might reduce the plasma concentration of lopinavir, ritonavir or sofosbuvir/daclatasvir, thus decreasing their therapeutic efficacy. On the other hand, vitamin E holds a beneficial drug interaction with ritonavir, as studies reported that vitamin E deficiency might aggravate ritonavir-induced hyperlipidemia through an increase CD36 expression [221]. However, to confirm the advantage of co-administration of ritonavir and vitamin E, additional in vivo trials are required.

No data from in vivo studies have been published to support the fact that vitamin E might affect intestinal drug metabolism. In guinea pigs administered with vitamin E for six weeks, the expression of the CYP3A4 protein was found to be unaltered [222]. Likewise, rats fed a diet high in vitamin E for two weeks had the same level of activity of the metabolic enzyme UGT1A1 as control animals [223]. A liver-specific transporter called organic anion transporting polypeptide (OATP) is known to facilitate the transport of different medications to the liver from the blood. There are conflicting studies on the effect of vitamin E on organic anion transporter 1 (OAT1) activity. Some studies demonstrated that feeding guinea pigs a vitamin E-rich diet for six weeks in the presence or absence of the OATP substrate had no effect on hepatic OATP expression or activity [222]. In contrast, a study found that rats given large doses of vitamin E had lower mRNA levels of hepatic OATP than the control group. Favipiravir, an RNA-dependent RNA polymerase inhibitor, is used to treat COVID-19 as a repurposed medication [224]. Additionally, the prodrug favipiravir is metabolized to an inactive metabolite and eliminated in the urine. Additionally, favipiravir inhibits OAT1 and OAT3, which aid in the kidneys’ ability to excrete uric acid [225]. Therefore, the inhibition of OAT1 with favipiravirquestions the concomitant use of this drug with vitamin E, but further research is required to obtain a firm conclusion about the risk of taking vitamin E and favipiravir together.

It is also worth mentioning that vitamin E is known to alter the metabolism of vitamin K as they are metabolized through the same pathway [226,227]. In agreement with that, studies have concluded that vitamin E decreased blood coagulation and increased bleeding tendency in human patients receiving vitamin E simultaneously with aspirin or warfarin [228]. Although it appears that vitamin E might hold an advantage for COVID-19 patients, especially against thromboprophylaxis, it still holds a serious drug interaction if co-administered with other anticoagulants such as heparin. In other words, COVID-19 patients taking heparin should be warned of bleeding tendency if co-administered with vitamin E. On another note, corticosteroids have been used as anti-inflammatory agents in COVID-19 therapy. A study has concluded that co-administration of vitamins D and E can improve the effectiveness of oxygen-dependent phagocytosis mechanisms and inhibit immunosuppressive effects of prednisolone [229]. The latter study proposes a clear interaction between corticosteroid administration and vitamin E.

## 9. Expert Opinion, Conclusions and Future Perspectives

Despite the immense efforts carried out by scientists for the discovery of new therapeutic modalities for treating COVID-19, and the evidence-based beneficial role of fat-soluble vitamins in preventing the cytokine storm associated with the severity of COVID-19, little is still known about the vitamin–drug interactions of fat-soluble vitamins and newly FDA-approved COVID-19 medications. Additionally, the pharmacodynamic and pharmacokinetic changes caused by these vitamins might alter the metabolism of COVID-19 therapeutic drugs. Such interactions could have negative consequences and hinder patients from taking the full therapeutic benefit out of the new modalities, particularly immunocompromised patients such as cancer and autoimmune patients. Thus, this review highlighted the critical vitamin–drug interactions between fat-soluble vitamins and the FDA-approved COVID-19 treatments and focused on personalized COVID-19 treatment protocols for cancer and autoimmune patients. This will help weigh the risks and benefits when tailoring personalized management protocols to such high-risk patients. Nevertheless, further research must be carried out to decipher the possible vitamin–drug interactions to maximize the benefits of these vitamins and prevent negative adverse events. Such information would be of great use by clinicians who will apply the concept of personalized medicine and implement personalized protocols and guidelines for preventing vitamin–drug interactions, particularly for high-risk patients such as autoimmune and cancer patients.

Currently, there are more than 200 clinical trials investigating the supplementation of vitamins in COVID-19 infection (https://clinicaltrials.gov/ct2/results?term=vitamins&cond=COVID-19 (accessed on 15 October 2022). Only one clinical trial (NCT04709744) focused on the impact of vitamin D supplementation on SLE patients during the COVID-19 pandemic. However, to date, no results have been posted or published from this clinical trial. Therefore, more clinical trials are needed to study the vitamin–drug interactions in order to draw a firm conclusion on the use of these vitamins during the course of COVID-19 infection. Additionally, studies are needed to study the drug–drug interactions between chronic medicines given to cancer/autoimmune patients and novel COVID-19 therapeutic modalities. This is in addition to exploring the prophylactic roles of these fat-soluble vitamins in COVID-19 infection, especially in immunocompromised patients. Furthermore, clinical studies should address the interactions between the forementioned vitamins in COVID-19-vaccinated individuals and specifically autoimmune and cancer patients.

## Figures and Tables

**Table 1:** Stratification of cancer patients with COVID-19 risk assessment upon infection.

Status of Cancer Patients	COVID-19 Risk
Undergoing chemotherapy	High risk
Performed surgeries	High risk
Prior cancer history	High risk
Smoking history	High risk
Undergoing immunotherapy	High risk
Undergoing radiation therapy	Low risk

**Table 3:** Therapeutic strategies used by cancer patients during the pandemic.

Cancer Type	Personalized Protocol	References
Breast cancer	Antiviral combination of darunavir/cobicistat in addition to tazobactam, piperacillin, levofloxacillin and hydroxychloroquine	[63]
Lung cancer	Combination of Hydroxychloroquine + azithromycin + lopinavir/ritonavir	[64]
Chronic lymphocytic leukemia	Hydroxychloroquine, Remdesivir, Lopinavir/Ritonavir, Tocilizumab, Intravenous immunoglobulin, Corticosteroids, Azithromycin, Convalescent Plasma	[65]
Solid and hematological malignancies	Azithromycin + Hydroxychloroquine	[66]
Multiple cancers	Azithromycin, Remdesivir, Tocilizumab, Hydroxychloroquine, Convalescent plasma, Systemic corticosteroids	[67]
Pediatric cancers	Broad-spectrum antibiotics, Hydroxychloroquine, Lopinavir/Ritonavir, Oxygen support	[68]

## Data Availability

Not applicable.
